# Effects of Copenhagen Adduction Exercise on the Architectural Characteristics of Adductors in U-17 Male Soccer Players: A Randomized Controlled Trial

**DOI:** 10.3390/ijerph182412956

**Published:** 2021-12-08

**Authors:** Alejandra Alonso-Calvete, Miguel Lorenzo-Martínez, Alexis Padrón-Cabo, Ezequiel Rey

**Affiliations:** 1 Facultade de Ciencias da Educación e do Deporte, Universidade de Vigo, 36310 Pontevedra, Spain; alejalonso@uvigo.es (A.A.-C.); miguel.lorenzo.martinez@uvigo.es (M.L.-M.); zequirey@uvigo.es (E.R.); 2 Department of Physical Education and Sport Science, Faculty of Sports Sciences and Physical Education, University of Coruña, 15001 A Coruña, Spain

**Keywords:** groin problems, injury prevention, muscle adaptation, ultrasonography, soccer

## Abstract

Groin injuries are one of the most prevalent in male soccer players, especially due to the hip adductor muscles’ weakness which is considered as a risk factor in these injuries. The Copenhagen adduction (CA) exercise has been demonstrated to increase the strength of adductor muscles, but its effects on the architectural characteristics of adductor muscles have not been studied yet. This study aimed to analyze the effects of the CA exercise on the muscle thickness of the adductors. Twelve male U-17 soccer players were randomized into two groups: the control group with no intervention and the experimental group with an intervention based on an eight-week training with CA exercise. The muscle thickness of adductors was measured before and after the intervention using ultrasound imaging. A significant increase in muscle thickness was found in both control (*p* = 0.002) and experimental group (*p* < 0.001), but the experimental group did not show additional effects in comparison with the control group. In conclusion, an 8-week CA exercise intervention does not increase the muscle thickness of adductors in U-17 soccer players more than their regular training.

## 1. Introduction

Soccer is a team sport with one of the highest injury rates [1] which is probably due to the frequent rotation and cutting movements [1,2]. In this sense, groin injuries represent in male soccer players one of the most prevalent, accounting for 7% to 13% of all time-loss injuries [2], with an incidence between 0.2 and 2.1/1000 h [2]. In this sense, hip adductor muscle injuries are the most common groin injuries in soccer players [3], constituting 64% to 69% of all groin injuries and causing a mean absence from sport of 15 days [4], resulting in substantial economic cost for clubs each season [5].

A groin injury is a complex and multifactorial problem, and previous studies have reported its variable etiology [6,7,8]. However, there is consistent evidence to suggest that the weakness of the musculature of the groin region, particularly reduced hip adductor and abductor strength, has been identified as one of the most important intrinsic risk factors for groin injuries [9,10]. Specifically, a decreased level of hip adductor strength may entail reductions in muscle capacity, asymmetries, and imbalances between hip adductor and abductor muscles, and an increase in injury risk during side-to-side cutting movements, changes of directions, or quick accelerations or decelerations [10,11]. All of these situations are widely prevalent in soccer, both in training and matches. For these reasons, exercise interventions to improve hip muscle strength seem to be important concerning prevention strategies, particularly in youth soccer players, trying to prevent groin problems in the future [6,12].

The Copenhagen Adduction (CA) exercise [13] is a non-equipment eccentric training exercise designed to strengthen both the hip adductor and abductor’s muscles and to optimize asymmetries between these muscles [14]. The CA has demonstrated significant adduction and abduction strengths improvements after standardized protocols when compared with a control group in soccer players [12,14,15]. Specifically, this exercise is effective in male elite soccer players [12] and also in male U-19 soccer players [14], demonstrating in both samples an increase of the strength in adductors, abductors, and the ratio between them. Moreover, the CA exercise has been suggested to be included in the FIFA 11+ training, which has been designed as a prevention program for soccer players to decrease injuries and especially the groin injury rate [15]. In addition, a recent cluster-randomized controlled trial showed reductions in the rate of groin injuries by 41% after an adductor-strengthening program based on CA in semi-professional soccer players, confirming its preventive effect [16]. However, the effects of CA on other variables like the architectural characteristics of the muscles have not been investigated yet.

In this regard, the use of ultrasound imaging has proven to be valid and reliable to assess the muscle architecture in the lower limb, not only in cadavers [17] but also in the architectural characteristics of rectus femoris [18] and biceps femoris [19] of healthy subjects. In this respect, several previous studies have used ultrasound imaging to assess changes in muscle architecture after preventive exercise programs like Nordic hamstring strength training [20,21] and reverse Nordic hamstring strength training [22], proving the ability of ultrasound imaging to identify structural muscle changes after preventive exercise training programs. In these investigations, the most studied variables were the cross-sectional area and the muscle thickness [20,21,22]. All of them reported differences before and after the eccentric training, and especially muscle thickness has been shown as one of the most studied, with high validity and reliability in the lower limb [20,21,22].

However, to the best of the author’s knowledge, the architectural characteristics of adductors have never been evaluated by ultrasound imaging. Considering the importance of the adductor muscles in the soccer practice and the high injury rate in this area, this study aimed to analyze the effects of an 8-week training with CA exercise on the architectural characteristics of adductor muscles, in youth soccer players (U-17), measured by ultrasound imaging. The main hypothesis was that the training program based on CA exercise would change the architectural characteristics of the adductors by increasing the muscle thickness after 8 weeks of training, in comparison with pre-test measurements and both legs.

## 2. Materials and Methods

### 2.1. Participants

Twelve youth male soccer players from the same U-17 regional soccer team were voluntarily recruited for this study. Exclusion criteria were injuries resulting in loss of one or more soccer matches/training sessions in the preceding 3 months before the initiation of the study. The players regularly performed 3 weekly soccer training sessions with their team in their normal training cycle. The team also regularly competed in one official match per week. None of the players had previously participated in a periodized training program using CA. All players and their parents were informed of the purpose of the study and gave their informed consent. This study was approved by an ethics committee and conducted according to the Declaration of Helsinki (2004). The subjects were randomized by a co-author not directly involved in testing or training into the CA group (*n* = 6) or the control group (*n* = 6). The randomization was computer generated.

### 2.2. Measures

This study lasted 10 weeks. In the first week, each subject completed 1 familiarization session based on the training CA exercise, to ensure its proper execution, and the pre-test measurements were assessed. Then, participants were randomized into two groups, intervention, and control group. In the intervention group, players performed CA exercises 2 times a week besides their regular training sessions, and in the control group participants only performed their regular training sessions. The protocol lasted 8 weeks, and in week 10, the post-test measurements of adductor muscle architecture were assessed. All data were collected at the same time of the day and under the same conditions to minimize the effect of diurnal variation.

### 2.3. Design and Procedures

A randomized controlled trial was conducted to analyze the effect of CA training on adductor muscle architecture.

Architectural parameters of the lower limb muscles have been studied previously by ultrasonography [21,22]. Specifically, muscle thickness has been demonstrated to have high reliability and applicability [18,19,23] in hamstrings and quadriceps. Nevertheless, the complex anatomy of the adductor muscles and their difficulty in measuring them separately at insertion has been reported [24]. Consequently, the adductor longus was chosen to be measured as it is the most superficial adductor and the main involved in groin injuries [24,25].

Ultrasound imaging has several advantages as its portability, the absence of radiation, high resolution of neuromuscular structures, and the ability to perform dynamic explorations [26]. Ultrasonography examination was performed with a 5–10 MHz lineal ultrasounds transducer (SonoSite M-Turbo^®^; FUJIFILM, Bothell, Washington, EEUU). All measurements were conducted and analyzed by a physical therapist with knowledge and qualification in ultrasound examination and trained in muscle thickness measurements (A.A.C.).

The examination was performed after 10 min of inactivity. Athletes were placed in the supine position with their dominant leg in flexion of hip and knee, and with external rotation in the hip [27]. First, pubic symphysis was located and marked as the insertion of adductor longus. Then, the skin was covered with a conductive gel and the probe was placed longitudinally to the muscle insertion and subsequently came down until the muscle belly appeared on the screen [24,25]. Finally, the image was frozen and the muscle thickness of adductor longus was measured, obtaining data in centimeters (cm). All measurements were assessed with minimal pressure in order not to alter the accuracy of the measurements [28].

After pretesting, the subjects in the CA group began an 8-week training program based on CA exercise (Table 1) in addition to the usual soccer training. The training program took place during the in-season period and was adapted from previously published programs [14,15]. CA is an eccentric exercise training for both the hip adductor and abductor muscles [14] that is performed in pairs [15]. The player started on the floor in a lateral position, only supported by his lower arm and foot. The upper arm stayed along the body and upper leg was handled by the partner on the ankle and the knee, and the body was raised in a straight line [14]. The exercise consisted of raising the lower leg, performing hip adduction, and then returning to the initial position gradually with no side flexion of the trunk in any part of the exercise [14]. During all CA training sessions, the correct performance was carefully supervised by the strength and conditioning coach, with experience in the exercise.

### 2.4. Statistical Analysis

All statistical analyses were carried out by SPSS for Macintosh (version 25.0, Chicago, IL, USA). The normality of the data was analyzed both graphically and with the Shapiro–Wilk test. The comparison between pre and post-test measurements was performed in both intervention and control group by a repeated-measure ANOVA design (MANOVA), with an inter-subject factor (group) and an intra-subject factor (moment of the measurement). The effect size was calculated using Partial eta squared (*η_p_*^2^). An effect of *η_p_*^2^ ≥ 0.01 is small, ≥0.059 is medium, and ≥0.138 is a large effect [29]. Pair-wise comparisons between the two different groups and the right and left legs were conducted via Bonferroni post-hoc test, using Cohen’s *d* to calculate effect sizes. These effects were classified as trivial (*d* ≤ 0.2) small (0.2 < *d* < 0.5), medium (0.5 < *d* < 0.8), and large (*d* ≥ 0.8) [29]. For all analyses, the significance value was set at *p* ≤ 0.05.

Reliability tests were also performed with 3 measurements of muscle thickness in both legs, obtaining both intraclass correlation coefficient (ICC) and coefficient of variation (CV) [30]. An ICC ≥ 0.7 indicated satisfactory reliability, ≥0.75 good reliability, and ≥0.9 excellent reliability [31]. According to previous reliability studies, a CV of less than 10% was set as the criterion for reliability [31].

## 3. Results

The test-retest reliability results for ultrasound imaging of adductor longus are shown in Table 2. For both legs, ICC values showed excellent relative reliability and CV confirmed correct absolute reliability.

Absolute values for each parameter at pre-test and post-test, together with the analysis of variance results are displayed in Table 3.

No significant time x group interactions were observed regarding muscle thickness for muscle thickness in right and left leg. The statistical analysis revealed a significant main effect of time in the right and left leg, indicating that muscle thickness increased in both groups and both legs from pretest to post-test. Finally, no significant main effect for groups was detected. In the pairwise comparisons, significant differences were found in the control group between pre-test and post-test measurements in the left leg (*p* = 0.002; *d* = 0.93, large) and in the right leg (*p* = 0.003; *d* = 0.91, large). In the pairwise comparisons in the intervention group, significant differences were found between pre-test and post-test measurements in the left leg (*p* = 0.002; *d* = 2.05, large) but not in the right leg (*p* = 0.063; *d* = 0.9, large).

## 4. Discussion

This study aimed to analyze the effects of an 8-week training program based on CA exercise in the muscle thickness of adductor longus, measured by ultrasound imaging, in a sample of U-17 soccer players. To the best of the author’s knowledge, this is the first study assessing the muscle thickness of adductor longus in a youth sample, and despite ultrasound imaging has been proven to be reliable in these measurements, it has been decided to calculate the reliability of this technique with the ICC and CV in the muscle selected, to provide more consistent results.

The main findings were: (a) an 8-week training program based on CA does not produce additional effects than the regular training in the muscle thickness of adductor longus in comparison with a control group in U-17 soccer players; and (b) a significant increase in the muscle thickness of adductor longus in both groups was observed.

Adductors’ weakness has been reported as one of the main risk factors of groin pain and groin-related injuries [7,10]. Therefore, strategies that aim to improve players’ strength levels may reduce such risk. In this respect, previous research reported significant improvements in hip adduction and abduction strength [5,14,15] and prevention of groin problems [16] after the implementation of CA-based training programs. However, this prior research analyzed adductor muscles globally through strength tests, which makes it difficult to know the real implication of each adductor in the global improvement. Considering that each muscle belly of the adductors presents architectural differences, their implications in strength or the injury incidence could be different too [24]. The present study analyzed only adductor longus as it is the main adductor related to groin pain and the one which provides more reliable measures [25]. Nonetheless, a global analysis of adductor muscles’ thickness could provide different results and show differences between the adductor muscles in terms of muscle thickness. Moreover, ultrasound imaging has proved to be a useful tool in the evaluation of musculoskeletal injuries since it enables a dynamic exam of the area and provides high-resolution images of the neuromuscular tissues [26]. Consequently, the study of the architectural characteristics by ultrasound imaging could be useful in studies of incidence, similar to Harøy et al. [16], providing valid and reliable information which could be used to detect in-depth changes in the tissues, such as small injuries or muscle scars [16].

This is the first study assessing the architectural characteristics of adductor muscles after a CA exercise program. The results of this study showed a large training effect on the muscle thickness of the adductor longus in both the right and the left leg. The increase of the muscle thickness was similar in both legs, with no significant differences between them. For this reason, it could be concluded that there were no significant benefits of adding CA exercise to the regular training routine of a team of youth soccer players. However, it is hard for this finding to be put into perspective with the literature, as no other study has investigated the effect of a CA training program on architectural characteristics. Nevertheless, prior research has evaluated architectural variables after programs based on other preventative eccentric exercises, such as the Nordic hamstring exercise or the reverse Nordic hamstring exercise, obtaining improvements in the muscle thickness of rectus femoris [22] and other architectural characteristics in semitendinosus [20] and biceps femoris [21]. Even though CA exercise and Nordic hamstring are both eccentric exercises, the complex insertion and distribution of adductor muscles [24] and their frequent injury and reinjury, mostly caused by a large use of these muscles in soccer training and matches [32], could also explain the different results in architectural characteristics between CA and Nordic hamstring. Specifically, the relationship between adductors’ insertion and pubic symphysis sometimes results in adductor tendinopathy or symphyseal degeneration, which may affect the normal contraction of the adductor muscles [25].

Another possible explanation for the absence of additional effects of CA exercise could be in relation to the sample used in the present study, composed of U-17 soccer players. This age category and its maturational status are characterized by rapid increases in muscle mass as a result of increasing sex hormone concentrations [33]. Thus, the predominantly hormonal training adaptations at this stage could influence the results obtained, and regular training sessions could be enough to induce changes in muscle thickness, justifying the absence of additional advantages of CA exercise. This fact is controversial as it has been mentioned that preventive exercises should be included in the training routines from the first stages of the sports. Especially in soccer, the high injury rate in the groin area, related in most cases to the weakness of the adductor muscles, makes the CA exercise a great exercise to fight against these injuries. Therefore, probably an 8-week training program based on CA exercise could be enough for elite soccer players, but other distribution of the sets and, presumably, more weeks of training with CA exercise may be needed for youth soccer players to achieve the same results as adults.

Furthermore, this study has several limitations which should be mentioned. First, the sample size comprising 12 soccer players, 6 per group, and all of them from the same team, and with short experience in strength and conditioning exercises makes it difficult to show differences between both groups. Second, the muscle thickness was measured in adductor longus, but other architectural variables could provide different results and other adductor muscles could show different changes. Third, measuring other variables such as the strength or the activity of the muscle could make these results more consistent.

Exercise selection is a major determinant of strength training adaptations. In this regard, CA is a dynamic, affordable, and easy exercise [14] and even though this study does not report changes in the muscle thickness of adductor longus, future investigations could take these findings into account to address in detail its advantages. Moreover, age seems to be a determining factor in this exercise, so future research could consider adjusting the CA programs to youth players, with different adaptations to exercise and evaluate their usefulness in studies with bigger sample sizes.

## 5. Conclusions

In conclusion, the muscle thickness of the adductor longus increased similarly in both the intervention and in the control group, with no differences between legs. Thus, CA exercise does not increase the muscle thickness of adductor longus in U-17 soccer players more than the regular training program.

## Figures and Tables

**Table 1:** Progression in training based on Copenhagen adduction exercise.

Week	Session/Week	Sets/Side
1	2	6
2	2	8
3	2	10
4	2	10
5–6	2	12
7–8	2	15

**Table 2:** Absolute and relative reliability.

	ICC (CI 95%)	CV (%)
**Left Leg**	0.97 (0.92–0.99)	2.54
**Right Leg**	0.96 (0.89–0.98)	3.19

**Table 3:** MANOVA test comparing pre and post-intervention in both groups.

	Control Group		Intervention Group		ANOVA *p*-Value (*η_p_*^2^)		
	Pre	Post	Pre	Post	Time	Group	Time x Group
**Left Leg**	0.536 ± 0.072	0.609 ± 0.084	0.541 ± 0.050	0.625 ± 0.029	<0.001 (0.766)	0.786 (0.008)	0.692 (0.016)
**Right Leg**	0.541 ± 0.082	0.617 ± 0.085	0.542 ± 0.050	0.591 ± 0.058	0.002 (0.625)	0.764 (0.009)	0.393 (0.074)
